# Efficient Terahertz detection in black-phosphorus nano-transistors with selective and controllable plasma-wave, bolometric and thermoelectric response

**DOI:** 10.1038/srep20474

**Published:** 2016-02-05

**Authors:** Leonardo Viti, Jin Hu, Dominique Coquillat, Antonio Politano, Wojciech Knap, Miriam S. Vitiello

**Affiliations:** 1 NEST, Istituto Nanoscienze – CNR and Scuola Normale Superiore, Piazza San Silvestro 12, Pisa, I-56127; 2 Department of Physics and Engineering Physics, Tulane University, New Orleans, LA-70118, USA; 3 Laboratoire Charles Coulomb (L2C), UMR 5221 CNRS-Université de Montpellier, Montpellier, France; 4 Università degli Studi della Calabria, Dipartimento di Fisica, via Ponte Bucci, 87036 Rende (CS), Italy; 5 Institute of High Pressure Institute Physics Polish Academy of Sciences Warsaw Poland.

## Abstract

The ability to convert light into an electrical signal with high efficiencies and controllable dynamics, is a major need in photonics and optoelectronics. In the Terahertz (THz) frequency range, with its exceptional application possibilities in high data rate wireless communications, security, night-vision, biomedical or video-imaging and gas sensing, detection technologies providing efficiency and sensitivity performances that can be “engineered” from scratch, remain elusive. Here, by exploiting the inherent electrical and thermal in-plane anisotropy of a flexible thin flake of black-phosphorus (BP), we devise plasma-wave, thermoelectric and bolometric nano-detectors with a selective, switchable and controllable operating mechanism. All devices operates at room-temperature and are integrated on-chip with planar nanoantennas, which provide remarkable efficiencies through light-harvesting in the strongly sub-wavelength device channel. The achieved selective detection (∼5–8 V/W responsivity) and sensitivity performances (signal-to-noise ratio of 500), are here exploited to demonstrate the first concrete application of a phosphorus-based active THz device, for pharmaceutical and quality control imaging of macroscopic samples, in real-time and in a realistic setting.

Two-dimensional (2D) van der Waals layered materials^1^,^2^ display an extraordinary technological potential for engineering nano-electronic and nano-photonic devices and components; they also provide an intriguing platform for fundamental investigations, through the exploitation of their confined electronic systems. If placed on chip with flat integrated optical circuits^3^, they can allow maximal interaction with light, therefore optimally utilizing their novel and versatile properties for a large number of applications in optical communications^4^, spintronics^5^, high-resolution sensing^6^ and tomography. Amongst the growing scale of required capabilities, the need for a photo-detection platform combining high speed, efficiency, wavelength range, flexibility, and integrability with more conventional semiconductor-based technologies is becoming more eminent^7^,^8^.

In the last few years, graphene and related materials have rapidly established themselves as intriguing building blocks for devising photo-detectors^7^: graphene exhibits ultrafast carrier dynamics, wavelength-independent absorption, tunable optical properties via electrostatic doping and high-mobility, which enables ultrafast conversion of photons or plasmons to electrical currents or voltages^9^. As a major distinctive characteristic, graphene is gapless, allowing charge carrier generation by light absorption over a very wide energy spectrum, while always conducting a significant amount of electricity. This inherent “leakage” can however significantly affect the efficiency of graphene-based devices.

Layered 2D materials with a band gap have recently triggered an increasing scientific interest^7^,^10^. Behaving like semiconductors, they only conduct electricity whenever the electrons absorb enough energy through heat, light, and other means. Depending on their specific band structures, these materials can disclose peculiar functionalities to be exploited for highly efficient light detection. With an energy gap in-between the gapless graphene and the larger gap transition-metal dichalcogenides, black phosphorus (BP) recently emerged as a fascinating and versatile material for photo-detection^3^,^11^.

BP is composed of stacks of its monolayer structure, phosphorene, along the *z* axis. Its band gap can be engineered and tuned by varying the number of layers stacked together^12^. Additionally, BP is a “direct-bandgap” semiconductor, meaning that it has the potential to efficiently convert electrical signals back into light. Unlike layered crystals with flat in-plane lattice, the BP monolayer, puckered along the armchair (*x*) direction, creates a fully anisotropic band structure that reflects in a large electrical, thermal and visible/near IR-optical in-plane anisotropy^12^,^13^,^14^,^15^. As a result, being an intrinsically flexible material that converts heat in energy with high efficiency and with performances that do not require any sophisticated engineering, BP can effectively provide new functionalities in a variety of device applications.

Despite the recent demonstration of high-performance waveguide-integrated^3^ or junction-based BP photo-detectors at telecom and mid-infrared frequencies^11^,^16^,^17^, the exploitation of BP for light detection at THz-frequencies is still at its early stage^18^, although the inherent material anisotropy can be appealingly exploited to devise detector architectures in which the photo-detection mechanism can be completely “designed”.

Photodetection of light, i.e. conversion of photons into a stable electrical signal, at THz frequencies can be accomplished by several different mechanisms like photo-thermoelectric, photovoltaic, galvanic, bolometric, plasma-wave rectification or via a combination of them^7^,^19^. Here, we engineer the architecture of antenna-coupled THz nanodetectors exploiting thin flakes of exfoliated BP, to selectively activate each of those processes, individually. The inherent electrical and thermal in-plane anisotropy of BP is exploited to selectively control the detection dynamics in the BP channel, at room-temperature (RT) and with state-of-the art detection efficiency.

## Results and Discussion

**Figure 1:**
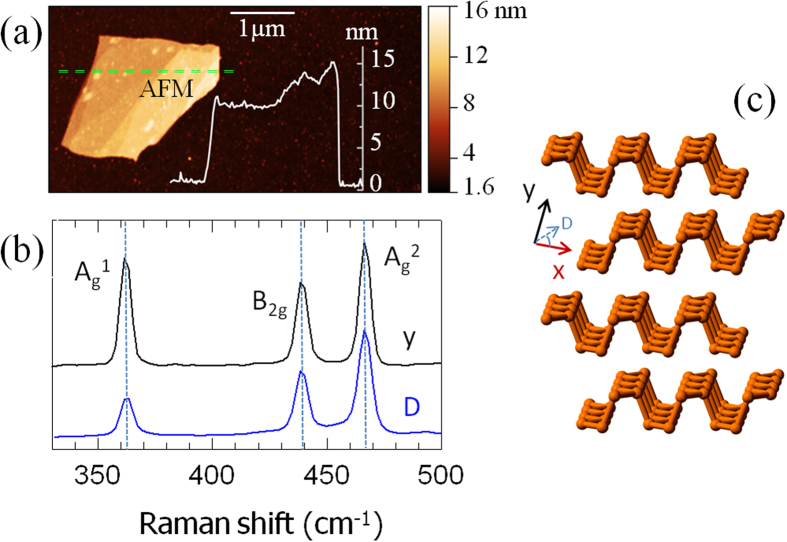
Flake identification and characterization. **(a)** Atomic force microscopy topographic image of an individual flake with thickness 15 nm. A topographic line profile, acquired along the dashed green line is shown. **(b**) Micro-Raman spectrum collected by exciting the samples along the z axis with the 532 nm line of a Nd-Yag pumping laser and by varying the polarization between the zigzag (y) and the 45° orientation angle (D axis) between the armchair (x) and y axis. Peaks are found at 362, 440, and 468 cm^−1^, corresponding to the A_g_^1^, B_2g_, and A_g_^2^ vibrational modes, respectively. **(c)** BP atoms are arranged in puckered honeycomb layers bounded together by Van der Waals forces; the armchair (x) and zigzag (y) crystal axis are shown on the graph.

Single-crystalline ingots of BP were grown via a chemical vapor transport technique similar to the one reported in ref. 20 (see Methods). Flakes having thickness in the range 8-14 nm were then mechanically exfoliated from bulk BP crystal using a standard adhesive tape technique on a 300 nm thick SiO_2_ layer on the top of a 300 μm-thick intrinsic silicon wafer. The exfoliated flakes were initially identified via optical and scanning electron microscopy (SEM) and then characterized with atomic force microscopy (AFM), Fig. 1(a), and linearly polarized micro-Raman spectroscopy, Fig. 1(b), to determine the layer thickness and the crystallographic directions, respectively (see Fig. 1(c) and Methods). Thin flakes with thicknesses *h* ~ 9 nm (corresponding to ~ 15 layers) and *h* ~ 14 nm (~ 23 layers) were individually contacted with proper adhesion layer/metal sequences to define the source (S) and drain (D) electrodes of a field effect transistor (FET) via aligned electron beam lithography (EBL) (see Methods). We fabricated two sets of samples: in sample A (23 layers) the S-D channel was defined along the *D* axis (see Fig. 1c), i.e. at a 45° angle in-between the armchair (*x*) and zigzag (*y*) directions; in sample B (15 layers) the S-D channel was conversely defined along the *y*-axis. Micro-Raman spectra shown in Fig. 1(b) (see Methods) provide clear indication of the channel orientation through the intensity ratio of the A_g_^2^ and A_g_^1^ active modes^13^.

**Figure 2:**
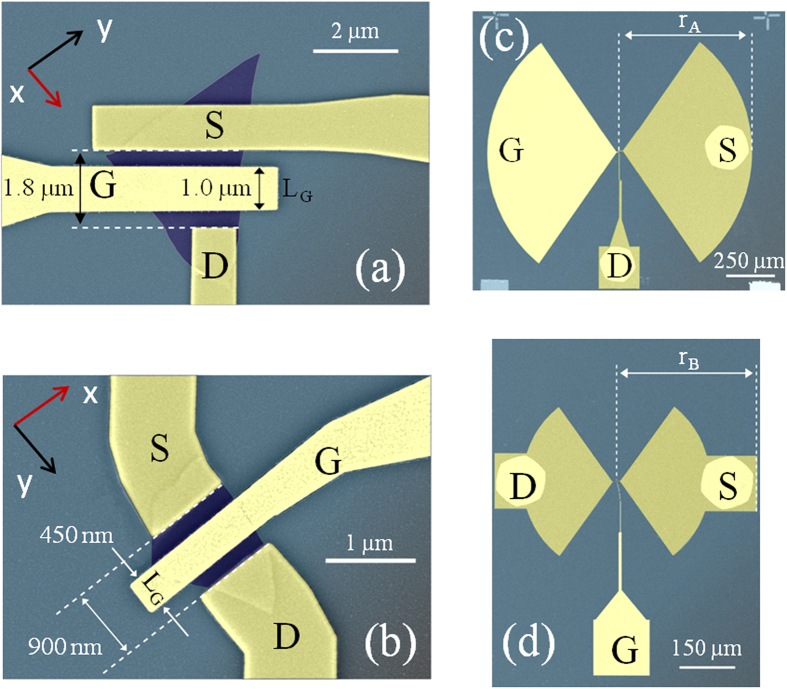
Device Fabrication. **(a,b)** False colors scanning electron microscope (SEM) images of the top-gated FETs in sample A (**a**) and sample B (**b**), respectively. **(c,d)** False colors SEM images of the patterned bow-tie antennas in sample A (r_A_ = 0.5 mm) and sample B (r_B_ = 0.25 mm), respectively.

In sample A, the S and gate (G) electrodes were then patterned in the shape of a half 110° bow-tie antenna, introducing a strong asymmetry in the mechanism of light harvesting; conversely, in sample B, a 110° bow-tie antenna was symmetrically placed at the S and D electrodes. In both cases, the antenna is designed to have maximum/resonant efficiency at 0.29 THz. The scanning electron microscopy (SEM) images of the device are shown in Fig. 2(a–d). The ratio between the channel length *L* and the gate length *L*_*G*_was kept ≤2, to reduce the portion of ungated channel regions as well as the related resistances and the parasitic capacitances.

**Figure 3:**
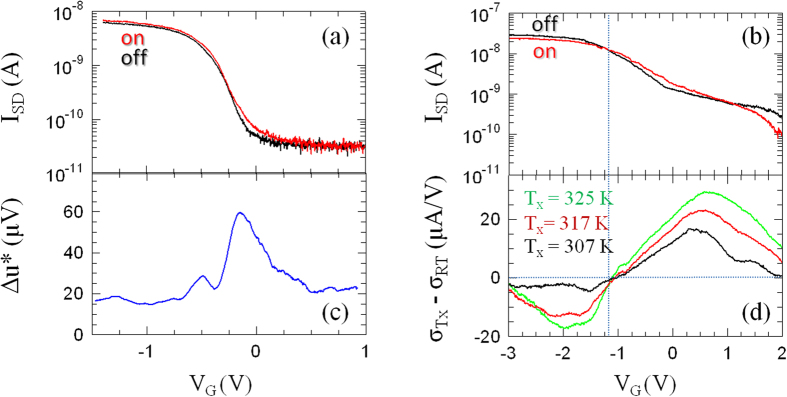
Transport characteristics. **(a,b)** Room-temperature (RT) transfer characteristics of sample A (**a**) and B (**b**) measured while sweeping V_G_, by keeping V_SD_ = 0.2 mV and while a 0.29 THz beam was impinging on the device (I_SD,on_) or when it was blanked (I_SD,off_). I_SD_ was amplified by a factor 10^6^ by using a transimpedance amplifier. **(c)** dc photovoltage trend vs V_G,_ measured from the difference between the I_SD,on_ and I_SD,off,_ multiplied by the channel resistance. **(d)** Gate voltage dependence of the difference between the RT conductance and the conductance measured at T_x_, in the three different cases in which T_x_ = 307 K, 317 K, 325 K. The dashed vertical line identifies the transition between the regime in which γ = dσ/dT < 0 (degenerate semiconductor) and γ > 0 (non-degenerate semiconductor).

Figure 3(a–b) shows the transconductance characteristics of sample A [Fig. 3(a)] and B [Fig. (3b)] measured while maintaining the gate bias (V_G_) below the breakdown voltage of the top-gate oxide (≈12 V for sputtered SiO_2_), and when the 0.29 THz beam of a tunable electronic source operating in the 0.265–0.375 THz range, was impinging on the device (I_SD,on_) or when it was blanked (I_SD,off_). All measurements have been performed along an identical gate-bias sweep direction, to rule out any possible hysteresis-related effect. The analysis of the transfer characteristics shows that the maximum device transconductance (g_m_) is partially affected by the BP flake orientation. Indeed, while the THz beam is blanked g_m_ = 9.1 nA/V in sample A and g_m_ = 19.0 nA/V in sample B. Conversely, when the THz light is funneled on the BP channel, g_m_ remains unchanged in sample A and decreases up to 11.5 nA/V in sample B, indicating a different detection mechanism. The analysis of the logarithmic plot of the transconductance curves also clearly unveils a large difference in the subthreshold slopes (S_s_)^−1^ which approach S_s_ = 238 mV/dec and S_s_ = 1136 mV/dec, for sample A and B (see Methods)^18^. The transconductance characteristics also provide a valuable way to extrapolate the carrier density (*n*) and the mobility (μ_FE_) through the relations *n* = C_gc_V_th_/(*e*·*h*·A_G_) and μ_FE_ = g_m_L_G_^2^/(C_TG_V_SD_), where A_G_ is the gated area and C_TG_ the total top-gate capacitance (see Methods). We extracted *n* ~ 8.0 × 10^18^ cm^−3^ and *n* ~ 2.2 × 10^19^ cm^−3^ for sample A and B, respectively. The mobility value is unaffected by the impinging THz light for sample A (*μ* ≈ 330 cm^2^/Vs). Conversely, a 30% THz light-induced carrier mobility reduction was found in sample B (*μ* ≈ 380–540 cm^2^/Vs).

In sample A, I_SD,on_ remains larger than the I_SD,off_ in the whole gate bias range, Fig. 3(a). The comparison between the two curves allows evaluating the *dc* photovoltage value [Fig. 3(c)] extracted from the measured photocurrent and labeled as 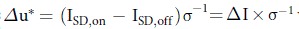 via the measurement of the overall channel conductance σ (extracted from Fig. 3a at device on), that provides a valuable way to predict the trend of the optical responsivity (R_ν_) and ac photovoltage (Δu). It is worth mentioning that ∆u* is however much less influenced by the loading than the photovoltage, being a purely *dc* measurement, not affected by the device capacitive reactance.

Conversely, sample B shows a peculiar V_G_-dependence of I_SD,on_ (Fig. 3b): for V_G_ < −1.1 V, I_SD,on_ < I_SD,off_, then in the range −1.1 V < V_G_ < 1 V, I_SD,on_ > I_SD,off_ and finally at V_G_ > 1, I_SD,on_ decreases below I_SD,off_, indicating three distinctive physical regimes. To better elucidate the nature of this behavior, we measured the conductance of sample B at three distinct temperatures T_x_, while heating it with an electric hot air gun and monitoring its temperature using a silicon diode thermometer in close contact with it. Figure 3(d) shows the voltage dependence of the difference between the RT conductance and the conductance measured at T_x_, in the three different cases in which T_x_ = 307 K, 317 K and 325 K. The plot clearly shows that the parameter γ = dσ/dT, which defines the bolometric coefficient, changes sign in correspondence of the first sign switch between I_SD,on_ and I_SD,off_ marked in Fig. 3(b). This means that BP behaves like a degenerate (γ < 0) and non-degenerate (γ > 0) semiconductor along its transconductance curve. Conversely, the sign switch at V_G_ = 1 V [Fig. 3(b)], not unveiled in the temperature-dependent plots of Fig. 3(d), can be likely ascribed to the THz-light induced voltage imbalance between the two antenna lobes, which adds to the static applied voltage V_SD_ = 0.2 mV.

**Figure 4:**
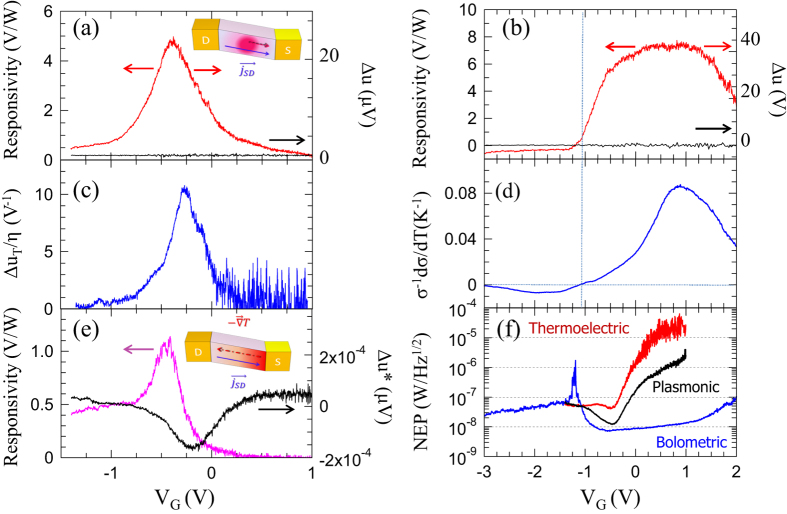
Terahertz detection. **(a,b)** Gate-bias dependence of the experimental RT responsivity (R_ν_)/photovoltage (Δu) for sample A (**a**) and B (**b**). The red lines were measured by impinging the 0.29 THz beam on the detectors surface; the black lines were collected while blanking the beam with an absorber. Inset: schematics of the overdamped plasma-wave dynamics. **(c)** Predicted photoresponse of sample A as a function of V_G_, under the overdamped plasma-wave regime. **(d)** Predicted bolometric trend, estimated from the gate voltage dependence of the ratio σ^−1^dσ/dV_G_ for sample B. **(e)** Left vertical axis: Gate bias dependence of the experimental RT responsivity of sample A collected while funneling the antenna resonant-frequency detuned 0.32 THz beam on the FET channel. Right vertical axis: dc photovoltage trend vs V_G,_ measured from the difference between the I_SD,on_ and I_SD,off,_ multiplied by the channel resistance. Inset: schematics of the thermoelectric dynamics **(f)** Noise equivalent power (NEP) as a function of V_G_, for the plasma-wave (sample A, 0.29 THz), thermoelectric (sample A, 0.32 THz) and bolometric (sample B) detectors extracted from the ratio between the thermal noise spectral density N_th_ = (4k_B_Tσ^−1^)^½^ and the device responsivity.

Figures 4(a,b) show the responsivity/photovoltage trends plotted as a function of V_G_ and measured, via a lock-in acquisition technique (see Methods), when a 0.29 THz beam was impinging on device A and B, respectively, with V_SD_ = 0 V.

In the case of fully asymmetric FET architectures, as in the case of sample A, two main effects are expected to be triggered by the incoming THz radiation: (*i*) the excitation of plasma oscillations along the FET channel^21^,^22^; (*ii*) the heating of the metallic contacts due to the THz-driven local currents inside the antenna arms^23^. By contrast, symmetric geometries, like the one employed for sample B, can likely induce bolometric detection effects, under specific material/geometry configurations. Both plasma-wave and thermoelectric effects are conversely prevented by the inherent symmetry.

The plasma-wave rectification effect, triggered by the antenna asymmetric radiation feeding in the conductive channel, results in an asymmetric charge density modulation, which will in turn induce a longitudinal electric field, with a preferential direction for the current flow. Under this regime (diffusive overdamped plasma-wave self-mixing regime), the generated photoresponse can be deduced from the transfer characteristics of the FET via the relation^21^,^22^,^24^:


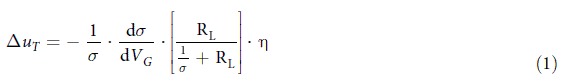


where *R*_*L*_ is the finite impedance of the measurement setup including the readout circuitry and the constant η represents the antenna-dependent coupling efficiency.

Equation (1), applied to the plot in Fig. 3(a), results in the predicted photovoltage trend shown in Fig. 4(c). The comparison with the corresponding experimental Δu curve [Fig. 4(a), right vertical axis], shows good agreement: R_ν_ peaks at negative V_G_ without any sign switch, as expected, and in full agreement with the predicted behavior.

Whenever a 2D semiconductor is non-uniformly heated, its thermal and electrical properties are locally modified and a steady state current density (I_T_) can be generated. The latter can be expressed as in ref. 19:


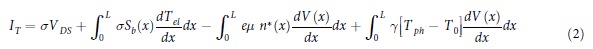


The first term is the steady-state *dc* current driven by the applied drain-to-source bias *V*_*SD*_; the second term is the photothermoelectric current (I_pe_) generated either by a temperature gradient within a non-zero Seebeck coefficient (*S*_*b*_) homogeneous material or by the electrical contact between two materials with different *S*_*b*_ (thermocouple junction)^14^,^22^; the third term is the photovoltaic contribution (*I*_*pv*_) induced by the excitation of carriers from the valence to the conduction band via interband transitions excited by the absorption of photons, (n^*^ is the density of photo-excited carriers); the last term takes into account the bolometric contribution (*I*_*B*_) to the current driven by the thermal excitation of the lattice (*T*_*ph*_ is the phonon temperature) that modifies the conductivity of the material through the bolometric coefficient γ, which depends from the strength of the electron-phonon coupling. The derivative *dV(x)*/*dx* here represents a static collection bias along the channel.

In the present case, by keeping in mind that the THz optical power is effectively transferred to the flake by the antenna, the following considerations can be done: i) the first term in (2) contributes to the dark current and it is negligible in our case, being V_SD_ = 0 V; ii) I_pe_ can play a role only for sample A; no temperature gradient is indeed present along the FET channel of sample B, as an effect of both the symmetric radiation feeding and the reduced distances between the metallic electrodes (heat-sink). Moreover, the thermoelectric signal generated by the metal-BP thermocouple junctions is zero, because the thermocouple current is always driven from the low S_b_ material (S_b_ ≈ 0 μV/K for metals) to the high S_b_ material (S_b_ ≈ 100 μV/K for BP, ref.^19^) and the contributions from the S and D sides of the channel are equally compensated. Furthermore, it is worth mentioning that, although the Seebeck coefficient is isotropic, the temperature gradient is expected to change in BP-FET with the orientation of the flake, being the thermal conductivity strongly anisotropic and larger (of ≈ a factor of 2.7, ref. 25) along the zigzag axis;^26^ iii) I_pv_ cannot be activated in our samples because the THz photons energy is much lower than the BP energy gap (≈ 300 meV for flakes thicker than 10 nm, ref. 13); iv) in the case of sample A, the asymmetric feeding of the radiation produces a not negligible temperature gradient across the FET channel. Thus, a strong thermoelectric response, caused by the induced diffusion currents, is expected within the BP flake. Due to the absence of a dc bias, the thermoelectric effect is expected to be strongly dominant with respect to the bolometric effect^19^. Conversely, the latter might play a non-negligible role in sample B, in which the radiation is symmetrically fed at the S-D electrodes; in this latter case the static voltage gradient dV(x)/dx along the FET channel is provided by the different boundary conditions at the S (grounded) and D (open-circuit) FET sides.

In the case of a degenerate semiconductor, *S*_*b*_ can be expressed through the Mott equation^18^, which has the same functional dependence set by Eq. (1), therefore preventing to discriminate between the thermoelectric and the plasma-wave effects, when the channel architecture and device geometry allows both detection processes to be activated. However, the comparison between Δ*u* and Δu* provides a valuable way to unveil the dominant effect that is contributing to the detection.

Under the assumption that thermoelectric effects dominate in our device A, the THz-induced carrier distribution gradient generates a diffusive flux of holes from the *hot*-side (S) to the *cold*-side (D) of the channel, hence, under zero-bias operation, (positive) charges will be accumulated at D whose potential will rise from zero to a (positive) thermoelectric value Δu_pe_. Conversely, if V_SD_ ≠ 0 (with V_D_ > V_S_ being S grounded) a certain amount of current will flow through the channel. If the sample is kept in the dark, the only electromotive force will be provided by the *dc* voltage V_SD_, and I_SD,off_ [Fig. 3(a)] will flow from D to S, i.e. in the opposite direction with respect to the light induced generated photothermoelectric current (I_pe_)^18^. Thus if I_SD,off_ > 0, Δu*_pe_ = (I_pe_-I_SD,off_)/σ will be negative while Δu_pe_ > 0, in clear disagreement with our experimental data [Figs 3(c), 4(a)] where both Δu and Δu* are positive.

On the other hand, if over-damped plasma-wave effects dominate in our device A, the excited carrier density would be pushed toward one channel side or the other depending on the structure asymmetry. In fact, at zero bias (V_SD_ = 0 V), the sign of the plasma-wave photovoltage Δu_pw_ is not known a priori. However, the corresponding Δu*_pw_ is well defined, since the applied *dc* voltage V_DS_ sets an asymmetric direction across the channel that will cause the charge density to drift towards the S side. The THz-induced current will then sum up with the pre-existent *dc* current, leading to Δu*_pw_ > 0, in agreement with our experimental data [Fig. 3(c)].

These considerations support the conclusion that sample A behaves like a plasma-wave THz detector, operating in the non-resonant overdamped regime, being ωμ*m*_d_^*^/e ≪ 1, where m_d_^*^ is the hole effective mass^27^ along the D-axis, *e* the electron charge and ω/2π = 0.29 THz^28^, which in the present experimental case/geometry means ν ≪ 0.85 THz.

The choice of a BP, *D*-axis oriented, flake is ideal to selectively activate the plasma-wave photodetection regime in sample A. The *x*-axis orientation, with its combined poor thermal conductivity and large electrical conductivity^12^, is indeed expected to enhance the BP thermoelectric performance while still allowing the activation of plasma-wave effects. Instead, the *y*-axis orientation can significantly reduce the sensitivity performance of the plasma-wave detector due to the major increase in the channel resistance^12^ (eq. 1).

The devised architecture allows the complete switching from a plasma-wave to a fully thermoelectric detection behavior. In the employed device configuration, the impedance matching between the device and the antenna is strongly frequency-dependent. Moreover, the generation of plasma-waves within the gated region requires a precise phase relation between the velocity and carrier density modulations for the rectification process to occur (see Methods). Therefore a slight change in frequency is in principle able to abruptly turn off the plasma-wave mediated THz detection. Figure 4e shows the photovoltage collected in sample A while detuning the frequency of the THz source (0.32 THz) from the antenna resonance (0.29 THz). In this latter case, the mechanism for light harvesting is not efficient enough to activate the oscillation and the mixing of the plasma-waves in the transistor channel. Therefore, the THz-induced thermal distribution gradient will generate a diffusive flux of holes from S to D, in opposite direction with respect to I_SD,off_. This sign discrepancy well reflects in the comparison between Δu* and the experimental responsivity curve [Fig. 4(e)].

To elucidate the nature of the detection process in sample B, we estimated the bolometric photovoltage (V_B_), via its functional dependence^19^ from the ratio 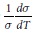:


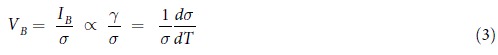


The trend reported in Fig. 4(d) is in excellent agreement with the experimental responsivity curve, thus confirming that device B behaves like a bolometer. Importantly, the responsivity curve in Fig. 4(b) shows a noticeable sign switch at V_G_ = −1.1 V, in perfect coincidence with the sign switch of γ [Fig. 4(d)], likely due to the huge carrier density modulation induced by the gate voltage. Furthermore, the bolometric photodetection mechanism, based on a light-induced change in conductance, is here due to the unveiled temperature dependent carrier mobility change [Fig. 3(b)].

The choice of the *y*-orientation is ideal for the activation of an efficient, single and selective bolometric detection process. Firstly, this ensures to fully suppress thermoelectric-related phenomena (strongly enhanced along the *x*-axis). More importantly, the longitudinal in-plane acoustic phonons show a sound speed (then a conductance) along the *y*-direction (8397 m/s) almost twice than the sound speed along the *x*-direction (4246 m/s). The thermal conductance is proportional to the squares of these sound speeds^12^. Conversely, the out-of-plane acoustic mode (ZA), which governs the electron-lattice cooling, exhibits a parabolic dispersion in layered materials and a significantly lower conductance. Thus, while an efficient heat-transfer from the antenna to the BP-flake is provided by the large in-plane *y*-axis thermal conductance, the electron cooling by acoustic phonons is slowed by the less effective e-ZA phonon coupling. This increases the device thermal resistance, which defines the bolometric sensitivity.

The responsivity curve in Fig. 4(b) shows the expected bolometric detection trend; when the conductivity of the flake is rather high (negative V_G_), the THz radiation is effectively absorbed, but the small relative change in absorption leads to a rather small responsivity; the highest responsivity is reached when the conduction of the flake is decreased and therewith the influence of the electron heating is maximized. When the conductivity is further decreased, the responsivity decreases again as the number of electrons available for intraband absorption is further decreased.

The background signal, measured while blanking the THz beam, is shown in Fig. 4(a) and Fig. 4(b) and allows extracting the signal-to-noise ratios (SNR): SNR ≈ 500 for the plasma-wave detector (sample A) and SNR ≈100 for the bolometric detector (sample B). By switching to a fully thermoelectric detection the SNR of sample A decreases to SNR ≈ 90. Remarkably, maximum R_ν_ of 5.0 V/W and 7.8 V/W have been reached for sample A and sample B, respectively, significantly larger than those reported in exfoliated graphene FETs^22^,^29^. The responsivity reduces to 1.1 V/W when device A operates in the thermoelectric regime. Under the assumption that the detector noise figure is dominated by the thermal Johnson-Nyquist contribution N_th_ = (4k_B_T/σ)^½^, we can infer the noise-equivalent power (NEP) i.e. the lowest detectable power in a 1 Hz bandwidth that can be calculated as N_th_/R_ν_^30^. Figure 4(f) shows the extrapolated NEP curves, whose minimum reaches 7 nW/√Hz, 10 nW/√Hz and 45 nW/√Hz for the BP-bolometer, plasma-wave and thermoelectric detector, respectively.

**Figure 5:**
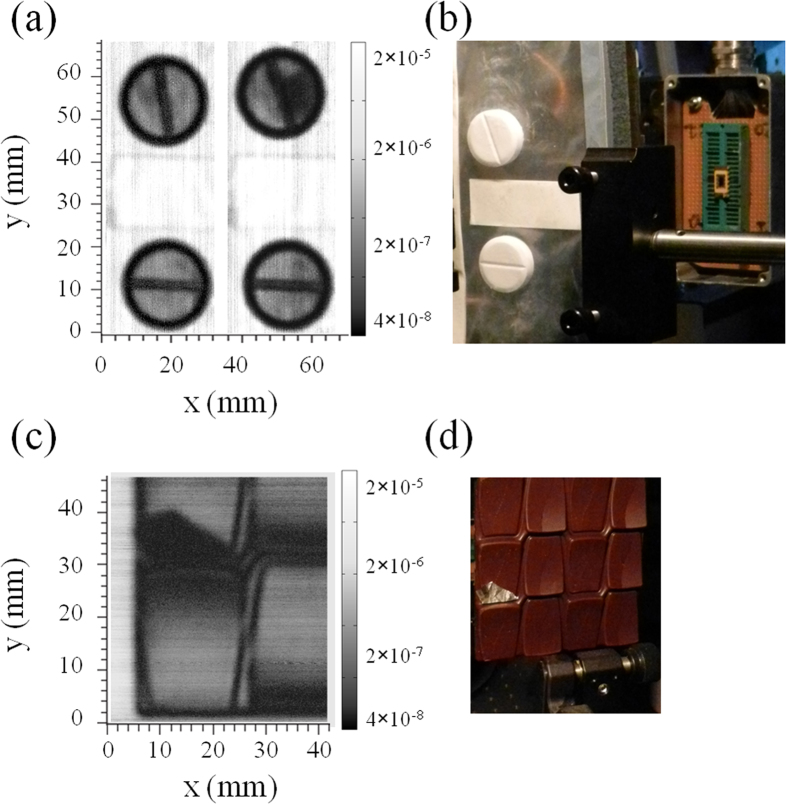
Large-area, fast, transmission THz imaging. **(a)** RT, transmission THz imaging obtained while impinging the 0.29 THz radiation on a box of two tablets before (left) and after (right) injecting 3 μl of water in one of them mounted on a *XY* stage, with an acquisition time of 10 ms/pixel. For visible light illumination the humidity-induced tablet deterioration, with the anomalous water content cannot be seen, either by naked eye or by the CCD camera used to take the related photograph **(b)**. The detection of THz transmitted radiation provides a visible signature on that. **(c)** RT, transmission THz imaging obtained while impinging the 0.29 THz radiation on a chocolate bar mounted on a *XY* stage, with an acquisition time of 10 ms/pixel. The residue of the aluminum external package is clearly resolved by the detectors. **(d)** Photograph of the chocolate bar collected from its rear side.

The remarkable SNR of the plasma-wave detector (sample A) has been exploited to provide concrete application examples of the devised detection technology, in a set of transmission imaging experiments. The 0.29 THz beam was focused on the target object and the transmitted power was detected in photovoltage configuration while keeping V_G_ = −0.4 V. The 400 × 700 pixel image of a set of target objects was acquired with a time constant of 10 ms. The THz scans are shown in Fig. 5(a,c) together with the correspondent photographs of the target objects. The transmission images [Fig. 5(a)] of a set of two tablets before (left side) and after (right side) injecting 3 μl of water in one of them, show that the BP detector clearly reveals the humidity-induced tablet deterioration, with the anomalous water content. Figure 5(c) shows the transmission image of a chocolate bar with some residue of the aluminum external package, clearly resolved by the BP detectors.

The versatility provided by the BP anisotropy and its tunable band gap unveils the potential of such material for optoelectronic and photonic devices with fully switchable response and performances, promising exceptional impact of few-layer phosphorene for devising active and passive THz devices and components.

## Methods

### Growth, material characterization and device fabrication

The BP single crystal was synthesized using a chemical vapor transport method^20^. The evacuated quartz tube containing red phosphorus has been placed into a double-zone tube furnace with temperatures set at 600 °C and 500 °C for the hot and cold end, respectively. Large-size single crystals of BP can be obtained after a week of transport.

The AFM mapping has been performed by employing a Bruker system (IconAFM) with thickness resolution <1 nm and lateral resolution ~20 nm. Each flake shows a layer thickness integer multiple of ~0.61 nm, i.e. the thickness of a BP monolayer (phosphorene).

Micro-Raman spectra have been collected by exciting the BP flakes of sample A and B along the z axis, by employing a Renishaw (InVia) system, equipped with a frequency doubled Nd:Yag 532 nm laser having maximum output power of 500 mW (CW). In the present experiments, we kept the optical intensity ≤0.4 mW/μm^2^, since intensity values larger than 0.8 mW/μm^2^ are likely to damage the flakes.

Thin flakes (*h* ~ 9–15 nm) were individually contacted via a combination of electron beam lithography (EBL) and thermal evaporation to deposit the (10/70 nm) (Ni/Au) S and D metal contacts. A 65 nm thick SiO_2_ oxide layer was then deposited on the sample via Ar sputtering, so that the exposed face of the BP-flake is fully encapsulated with it. This is a crucial step to prevent degradation due to ambient air humidity, that, conventionally, dramatically alter the electrical device performances and deteriorate the flake itself. The G electrode was aligned with the center of the channel via EBL and defined via thermal evaporation of 80 nm layer of Cr/Au. Sample A and B are equipped with antisymmetric (A) and symmetric (B) bow-tie antennas with radius *r*_*A*_ = 500 μm and *r*_*B*_ = 250 μm, respectively. For sample A, the channel length is *L* = 1.8 μm and the gate length is *L*_*G*_ = 1.0 μm while for sample B, *L* = 900 nm and *L*_*G*_ = 450 nm. Under this configuration, and in the presence of a 65 nm thick oxide layer, the simulated (3D FEM, COMSOL Multiphysics) geometrical gate-to-channel capacitance are C_gc_ ~ 1.44 fF and C_gc_ = 0.39 fF for samples A and B, respectively.

### Top-gate Capacitance estimation

For a thin layer channel, the top-gate capacitance (C_TG_) is usually expressed as:


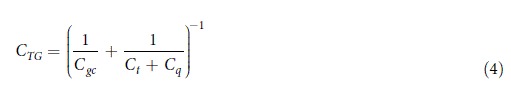


A significant portion of the electrostatic gate potential (V_G_) is dropped within the oxide to fill trap states (and interface states) and inside the channel to modify the carrier population. These two effects are typically modeled by introducing the interface trap capacitance (C_*t*_) and the quantum capacitance (C_*q*_). An estimation of the ratio C_t_/C_gc_ can be obtained from the subthreshold swing (S_s_) of the FET. S_s_ is conventionally expressed in terms of the band movement factor: β = [1 + (C_t_ + C_q_)/C_gc_].^12^ In the subthreshold regime, the channel is almost depleted of free carriers, hence C_q_ can be safely neglected, the channel bands move one-to-one with the applied gate bias. The logarithmic plot of the transconductance curve shows a linear region whose slope corresponds to the subthreshold slope (S_s_)^−1^. Being S_s_ ~ 60 β mV/dec, the linear fit to the data allows to retrieve S_s_ = 238 mV/dec and S_s_ = 1136 mV/dec, corresponding to β = 4 (C_t_ = 3 C_gc_) for sample A and β = 19, (C_t_ = 18 C_gc_) for sample B, respectively. The larger β value in sample B is fully consistent with the expected quantum capacitance increase in the thicker BP flake. By increasing V_G_, C_TG_ varies in the range [0.75 C_gc_, C_gc_] for sample A and in the range [0.95 C_gc_, C_gc_] for sample B.

### Optical characterization

The optical characterization has been performed by focusing the THz frequency beam on a spot of 4 mm diameter at the detector surface by means of a set of *f*/1 off axis parabolic mirrors and mechanically chopped at 619 Hz. The power of the source (P_t_), calibrated as a function of output frequency with a power meter, ranges between 200 μW and 400 μW. The responsivity was measured in a photovoltage-mode (PV) configuration: the S electrode was grounded, V_G_ was set with a Keithley *dc* generator and Δu was measured at the D electrode with a lock-in amplifier. A low-noise voltage pre-amplifier (input impedance = 10 MΩ) with a pass-band filter between 300 Hz and 1 kHz was used in the experiments with a gain factor G_n_ = 1000. From this voltage measurement Δu can be calculated using the equation^30^:


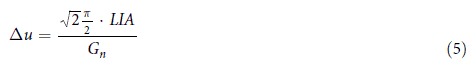


where *LIA* is the voltage read by the lock-in, and the factor π√2/2 is a normalization coefficient that takes into account that the lock-in measures the rms of the fundamental sine wave Fourier component of the square wave produced by the chopper. We preventively measure Δu as a function of frequency by employing the tunable source to identify the antenna resonances for sample A and B, that in both cases coincides with ν = 298.5 GHz (P_t_ = 300 μW). The intensity peak at ν_1_ = 324.6 GHz (P_t_ = 400 μW) provides a 70% response reduction. The responsivity (R_ν_) was then determined using the relation R_ν_ = (Δu·S_t_)/(P_t_·S_a_), where S_t_ is the beam spot area and S_a_ is the active area of the detector; in the case of sample A, S_a_ is set equal to the diffraction limited area (S_λ_ = λ^2^/4), being the antenna surface smaller than S_λ_^30^.

The total efficiency of the receiving antenna (η) can be approximated as the product of the collection efficiency ε_coll_, which represents the capability to collect a photon, and the mismatch efficiency ε_match_, which refers to the impedance matching between the antenna and the connected electrical circuit, i.e, as: η = ε_coll. _× ε_match_, where


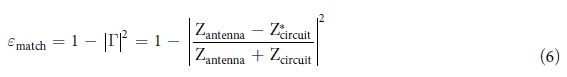


Here, Г is the reflection coefficient and Z_antenna_, Z_circuit_ represent the complex antenna and connected circuit impedances, respectively. When the antenna is operating at its resonance frequency, the imaginary part of Z_antenna_ drops to zero. ε_match_ is then a function of frequency and channel resistance (then gate voltage) in our experiments.

The minimum measured resistance of the BP flake is 20 kΩ, whereas the impedance of a planar bow-tie antenna is conventionally 70 Ω −100 Ω. Since in the case of a S-G antenna there is only a capacitive coupling between the circuit and the antenna, we can extract the capacitances and inductances playing a role in the FETs and then evaluate the complex impedance matching, which determines the electromagnetic power delivered to the BP flake. In the case of sample A, the maximum ε_match_ = 0.11, meaning that only 11% of the power collected by the antenna is delivered to the BP flake.

To assess the THz-induced photocurrent I_SD,on_ we exploited a photoconductive scheme: a *dc* bias (V_SD_) is applied to the source electrode and the current is read at the D electrode with an amperometer while shining the THz radiation on the detector.

## Additional Information

**How to cite this article**: Viti, L. *et al*. Efficient Terahertz detection in black-phosphorus nano-transistors with selective and controllable plasma-wave, bolometric and thermoelectric response. *Sci. Rep.* **6**, 20474; doi: 10.1038/srep20474 (2016).
